# Liver function markers predict cardiovascular and renal outcomes in the CANVAS Program

**DOI:** 10.1186/s12933-022-01558-w

**Published:** 2022-07-04

**Authors:** Giulia Ferrannini, Norman Rosenthal, Michael K. Hansen, Ele Ferrannini

**Affiliations:** 1 Department of Medicine Solna, Karolinska Institutet, Norrbacka, S1:02, 171 76 Stockholm, Sweden; 2 Janssen Research & Development, LLC, 920 US-202, Raritan, NJ 08869 USA; 3 Janssen Research & Development, LLC, Welsh & McKean Rds., Spring House, PA 19477 USA; 4 CNR Institute of Clinical Physiology, Via Savi 12, 56126 Pisa, Italy

**Keywords:** Alanine aminotransferase, Liver, Heart failure, Renal morbidity, SGLT2 inhibitor

## Abstract

**Background:**

Raised liver function tests (LFTs) have been correlated with multiple metabolic abnormalities and variably associated with cardiorenal outcomes. We sought to systematically test the relationship between LFT levels within the accepted range and major cardiorenal outcomes in a large clinical trial in type 2 diabetes, and the possible impact of placebo-controlled canagliflozin treatment.

**Methods:**

We measured serum alanine aminotransferase (ALT), aspartic aminotransferase (AST), gamma-glutamyl transferase (γGT), alkaline phosphatase (ALP), and bilirubin concentrations in 10,142 patients, at baseline and repeatedly over follow-up. The relation of LFTs to first hospitalized heart failure (HHF), cardiovascular (CV) and all-cause mortality, and progression of renal impairment was investigated using multivariate proportional-hazards models.

**Results:**

In univariate association, ALT was reciprocally predictive, and ALP was positively predictive, of all adjudicated outcomes; γGT also was directly associated with CV—but not renal—outcomes. In multivariate models including all 5 LFTs and 19 potential clinical confounders, ALT was independently associated with lower, and γGT with higher, CV outcomes risk. Canagliflozin treatment significantly reduced ALT, AST, and γGT over time. In a fully adjusted model including updated LFT levels and treatment, γGT was independently associated with CV and all-cause mortality, ALP with renal dysfunction progression, and canagliflozin treatment with significant reduction in HHF and renal risk.

**Conclusions:**

Higher γGT levels are top LFT markers of risk of HHF and death in patients with diabetes and high CV risk, while ALT are protective. Canagliflozin lowers the risk of HHF and renal damage independently of LFTs and potential confounders.

**Supplementary Information:**

The online version contains supplementary material available at 10.1186/s12933-022-01558-w.

## Background

Type 2 diabetes predisposes patients to serious complications, including atherosclerotic cardiovascular disease (ASCVD), heart failure (HF), and chronic kidney disease (CKD) [1, 2]. Such joint cardio-renal dysfunction increases morbidity, mortality, and healthcare costs [3, 4]. Recently, sodium-glucose cotransporter-2 inhibitors (SGLT2i) have proven beneficial in reducing the risk of ASCVD, HF, and renal outcomes in large cardiovascular outcome trials (CVOTs) [5–8]. Results from these trials rekindled attention to the pathophysiological interrelationships underlying these findings, and to accurate risk stratification of the comorbid, high-risk patients included in CVOTs [5].

Traditional so-called liver function tests (LFTs) abnormalities, including mildly elevated alanine aminotransferase (ALT), aspartic aminotransferase (AST), gamma-glutamyl transferase (γGT), alkaline phosphatase (ALP), or bilirubin, are well documented in patients with heart disease, reflecting passive hepatic congestion, reduced hepatic perfusion, or both [9]. The influence of renal function on LFTs and on their changes over time is more controversial and has been less investigated [10]. Altered LFTs (especially increased ALT) are frequently encountered in patients with type 2 diabetes as markers of hepatic steatosis and non-alcoholic fatty liver disease (NAFLD), along with markers of insulin resistance and dysfunctional adipose tissue [10–12]. Thus, LFTs correlate with the abnormalities occurring in the typical patient included in CVOTs, as they reflect cardiac, metabolic, and, possibly, renal dysfunction [13].

In CVOTs, however, LFTs usually only feature as safety monitoring tools, because drug-induced hepatotoxicity is the most common reason for drug withdrawal or limited use [14]. While a consensual, marked alteration of LFTs is an established indicator of liver dysfunction, little is known about mildly elevated LFT values and/or oscillating serum concentrations, and their association with features of cardio-renal dysfunction in type 2 diabetes [10, 12]. Indeed, mild increases in the serum levels of these markers might be due to other conditions, such as bone disease, muscle damage or hemolytic anemia.

A potential role of LFTs as predictors of incident CV events and mortality has been proposed, with contrasting results in studies including heterogeneous populations [13]. In brief, there is good evidence that increased γGT is associated with higher risk of CV and all-cause mortality, both in the general population and in individuals with type 2 diabetes [15, 16]. γGT in the upper reference range might reflect oxidative stress and cell damage, being associated with visceral fat accumulation, glutathione metabolism and low-density lipoprotein cholesterol (LDL-C) oxidation [17]. Increased bilirubin levels have been shown to be protective against future CV events, consistently in people with established CVD, but secondary analyses of the CHARM and PARADIGM-HF trials reported an opposite trend in patients with HF with reduced ejection fraction, possibly due to the predominance of the cholestatic pattern of LFT alterations [13, 18–20]. Elevated AST, ALT and ALP have been reported to be negative predictors of CV outcomes in population-based studies, but the available evidence is weaker compared with γGT and bilirubin, especially in patients with established cardiovascular disease (CVD) [13]. This could be due to the low specificity of transaminases levels in identifying non-alcoholic fatty liver disease as well as to other mechanisms causing ALP elevation, i.e., vascular calcification through increased bone metabolism and impaired calcium homoeostasis [13]. More recently, the AST/ALT ratio has been suggested to better reflect the extent of metabolic dysfunction, therefore being a more suitable marker in people with type 2 diabetes and a biomarker of CV disease and impaired renal function [10, 21].

These diverse and incomplete findings prompted us to test whether and which LFTs within the accepted range (i.e., < 3 × upper limit of normal, ULN) are independently related to major CV and renal outcomes adjudicated in a large placebo-controlled clinical trial, and what impact SGLT2i treatment may have on them. Thus, in the present exploratory analysis of the Canagliflozin Cardiovascular Assessment Study (CANVAS) Program [22], we systematically investigated the relation of LFTs to clinical phenotype, and cardiac and renal outcomes and death in patients with type 2 diabetes.

## Methods

Clinical data from the CANVAS Program are available in the public domain via the Yale University Open Data Access Project (http://yoda.yale.edu/). The trial protocols and statistical analysis plans were published along with the primary CANVAS Program manuscript.

The CANVAS Program integrated two trials, CANVAS and CANVAS-R, involving a total of 10,142 participants with type 2 diabetes and high CV risk. Details of the CANVAS Program design and oversight, participants, inclusion and exclusion criteria, randomization, treatment and follow-up, and outcomes have been published [22, 23]. Briefly, participants in CANVAS were randomly assigned in a 1:1:1 ratio to receive canagliflozin at a dose of 300 mg, canagliflozin at a dose of 100 mg, or matching placebo, and participants in CANVAS-R were randomly assigned in a 1:1 ratio to receive canagliflozin, administered at an initial dose of 100 mg daily—with an optional increase to 300 mg starting from week 13—or matching placebo. Use of background antihyperglycemic therapy and control of other risk factors were guided by best practice instituted in line with local guidelines. Adjudicated outcomes were major adverse cardiac events—MACE (a composite of death from CV causes, nonfatal myocardial infarction, or nonfatal stroke), death from any cause, death from CV causes, hospitalized heart failure (HF), the composite of death from CV causes and HF, and the renal composite outcome, comprising a > 40% reduction in estimated glomerular filtration rate (eGFR) sustained for at least two consecutive measures, the need for renal-replacement therapy (dialysis or transplantation), or death from renal causes (defined as death with a proximate renal cause), and progression to macroalbuminuria. Progression of albuminuria was defined as more than a 30% increase in albuminuria and a change from either normoalbuminuria to microalbuminuria or macroalbuminuria, or from microalbuminuria to macroalbuminuria.

The protocol for the CANVAS Program trial was approved by the local ethics committee at each trial site. All participants provided informed, written consent.

### Analytical methods

Serum ALT, AST, γGT, ALP, and bilirubin were measured at baseline and at week 6, 18, 39, 52, 78, 104, 130, 156, 182, 208, 234, 260, 286, 312, 338, and 364 post-randomization. Measurements were made centrally by Covance Central Laboratory using standardized procedures; for each LFT, sex- and age-specific ULN was used.

All available data were included in the analyses.

### Statistical analysis

Data are summarized as mean ± standard deviation (SD) or median [interquartile range (IQR)] for variables with a skewed distribution by the Shapiro–Wilk test; these latter variables were transformed into their natural logarithms for use in statistical analysis. Mean group values were compared by the Wilcoxon signed rank test and frequencies were compared by the χ^2^ test. Cumulative incidence of events is shown as Kaplan–Meier plots. Cox proportional hazards models were used to test the association of predictor variables with events; hazard ratios (HR) and their 95% confidence intervals (CI) were calculated for 1 SD for variables with a normal distribution and 1 log unit for variables with a skewed distribution. Initial univariate Cox models were run for the baseline (pre-randomization) value of the five LFTs. Adjusted models included all five LFTs as well as nineteen additional variables [sex, age, body mass index (BMI), baseline glycated hemoglobin A_1C_ (HbA_1c_), type 2 diabetes duration, investigator-reported history of HF, prior CV disease, cigarette smoking, urinary albumin-to-creatinine ratio (UACR), eGFR, systolic blood pressure (BP), LDL- and HDL-cholesterol, serum albumin, and use of diuretics, statins, antithrombotics, renin–angiotensin–aldosterone-system (RAAS) inhibitors, and ß-blockers]. In Cox models in which treatment with canagliflozin was added as a predictor, baseline LFTs values were replaced by their respective updated values, i.e., the average value up to week 104, at time at which at least 70% of the participants were censored. Time-course of LFTs by treatment were analyzed by 2-way ANOVA for repeated measures. A nominal *p* value ≥ 0.05 was considered significant. Analyses were performed using JMP 9.0.1^®^.

## Results

### Serum LFT levels

As shown in Additional file 4: Table S1, median LFT levels were well within the respective range, and very few subjects had levels exceeding 3*x*ULN on the day of the blood draw at baseline and at the 21-week visit, when > 70% of the participants were censored.

### Association between baseline LFTs and outcomes

**Fig. 1:**
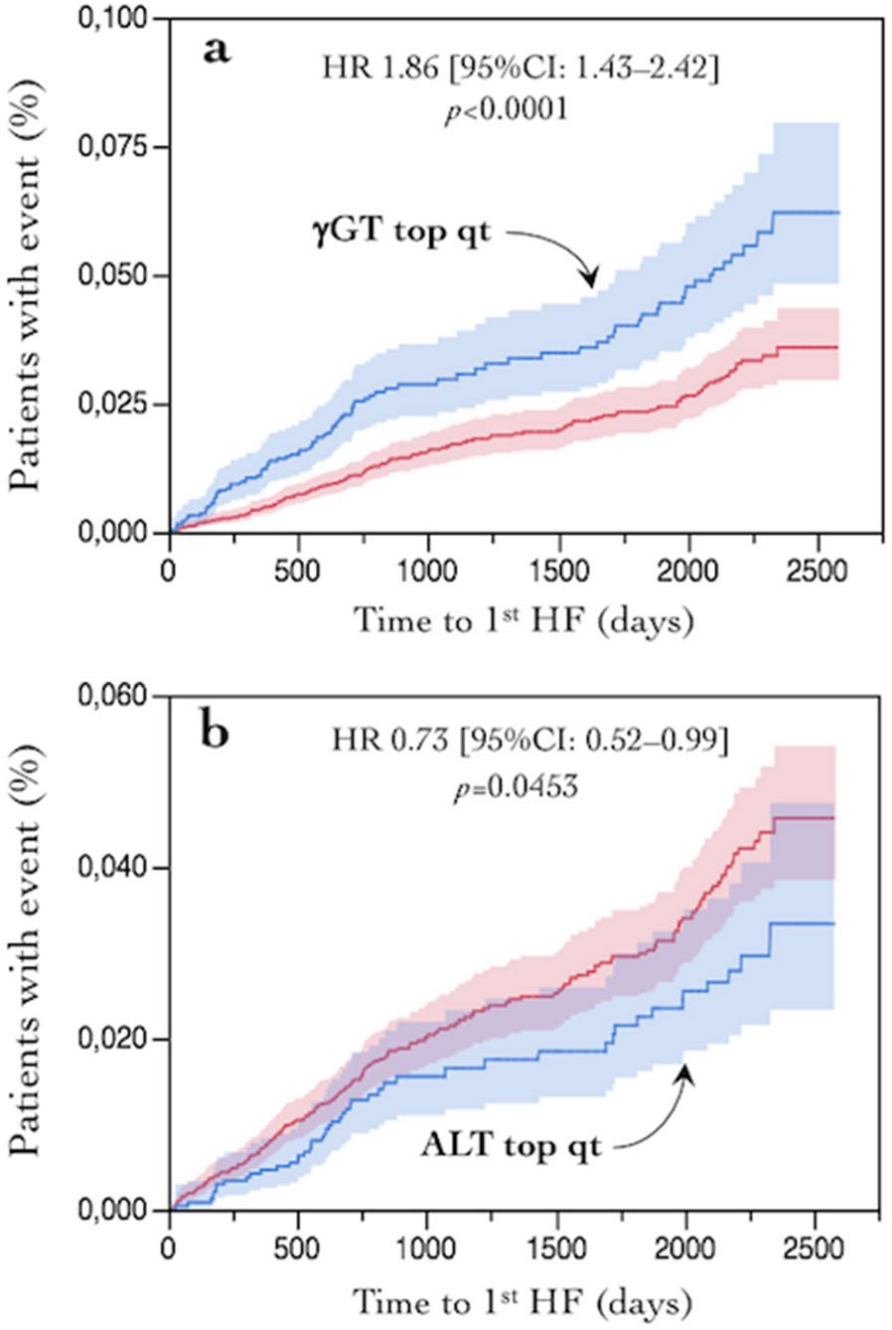
**a** Kaplan–Meier plot of time to first hospitalized heart failure by baseline serum gGT value (top quartile *vs* rest of the distribution); **b** Kaplan–Meier plot of time to first hospitalized heart failure by baseline serum ALT value (top quartile vs rest of the distribution)

Median follow up of the entire cohort was 2.4 (4.1) years. In univariate association, several baseline LFT values were associated with multiple outcomes—whether using the top quartile of their distributions or the log-transformed concentrations—but in opposite direction. For example, higher γGT predicted more HF, whereas high ALT predicted fewer, first hospitalizations (Fig. 1). Across endpoints, ALT was reciprocally predictive, and ALP was positively predictive, of all outcomes; γGT also was directly associated with CV, but not renal, endpoints, while bilirubin was inversely related to the renal endpoint (Table 1).

**Table 1:** Univariate association (HR [95% CI]) of individual baseline liver markers with CV and renal outcomes

	HF (n = 243)	CV death (n = 453)	HF or CV death (n = 652)	All deaths (n = 681)	MACE (n = 1011)	Renal endp.^a^ (n = 847)
Top quartile						
ALT	**0.73 [0.52–0.99]**	**0.59 [0.45–0.75]**	**0.63 [0.51–0.77]**	**0.64 [0.52–0.78]**	**0.84 [0.72–0.98]**	**0.75 [0.64–0.89]**
AST	1.04 [0.76–1.39]	0.80 [0.63–1.01]	0.89 [0.73–1.07]	0.89 [0.74–1.07]	0.99 [0.85–1.14]	0.94 [0.80–1.10]
ALP	**1.51 [1.15–1.98]**	**1.48 [1.20–1.81]**	**1.46 [1.23–1.72]**	**1.44 [1.21–1.69]**	**1.28 [1.11–1.47]**	**1.28 [1.10–1.48]**
γGT	**1.85 [1.42–2.40]**	1.03 [0.83–1.28]	**1.29 [1.09–1.53]**	1.18 [0.94–1.33]	1.10 [0.95–1.26]	1.06 [0.90–1.23]
Bilirubin	1.23 [0.93–1.63]	0.82 [0.64–1.02]	0.95 [0.78–1.13]	0.85 [0.70–1.02]	1.00 [0.86–1.15]	0.94 [0.80–1.10]
Log [baseline]						
ALT [U/L]	**0.62 [0.46–0.82]**	**0.52 [0.42–0.64]**	**0.55 [0.46–0.66]**	**0.55 [0.46–0.65]**	**0.84 [0.73–0.96]**	**0.72 [0.62–0.84]**
AST [U/L]	1.03 [0.71–1.47]	**0.64 [0.48–0.85]**	**0.75 [0.59–0.94]**	**0.74 [0.59–0.93]**	0.91 [0.76–1.09]	**0.80 [0.66–0.98]**
ALP [U/L]	**1.88 [1.24–2.83]**	**1.75 [1.29–2.38]**	**1.68 [1.30–2.17]**	**1.54 [1.20–1.98]**	**1.43 [1.16–1.75]**	**1.51 [1.21–1.89]**
γGT [U/L]	**1.65 [1.39–1.95]**	1.14 [0.99–1.31]	**1.30 [1.17–1.46]**	**1.13 [1.09–1.27]**	**1.17 [1.06–1.28]**	1.08 [0.98–1.20]
Bilirubin [µM]	1.07 [0.81–1.41]	0.87 [0.71–1.08]	0.94 [0.79–1.12]	0.85 [0.72–1.01]	1.02 [0.88–1.17]	**0.85 [0.73–0.98]**

Table entries are hazard ratio (HR) and 95% confidence interval. Statistically significant estimates are in bold

*ALT* alanine aminotransferase; *AST* aspartic aminotransferase; *γGT* gamma-glutamyl transferase; *ALP* alkaline phosphatase; *CV* cardiovascular; *HF* hospitalized heart failure; *MACE* major adverse cardiovascular events

^a^Renal endp. = composite of a > 40% decline in estimated glomerular filtration rate, renal replacement, renal death, and progression to macroalbuminuria

Baseline LFTs were all related to one another, the strongest associations being those between ALT, AST, and γGT, while ALP and bilirubin were less correlated with the other markers (Additional file 4: Table S2). In addition, LFTs were related to several anthropometric and biochemical characteristics. ALT and γGT, for example, were both directly related to male sex and BMI, and inversely related to age, whereas AST was a weak correlate of these characteristics; ALT and AST were significantly higher in individuals on statin therapy. The associations of serum albumin concentrations with LFTs were unexpected, however.

The multivariate association between LFTs and outcomes is reported in Table 2. γGT was a positive predictor of all CV outcomes and death but not of the renal endpoint, whereas ALT was a negative predictor of HF hospitalization, CV death, all-cause death and of the composite of HF hospitalization and CV death; AST, ALP, and bilirubin were not independently associated with any outcome.

**Table 2:** Multivariate association (HR [95% CI]) of all 5 baseline liver markers with CV and renal outcomes

	HF	CV death	HF or CV death	All deaths	MACE	Renal endp.^a^
Log [baseline]						
ALT [U/L]	**0.53 [0.34–0.84]**	**0.67 [0.47–0.97]**	**0.59 [0.44–0.79]**	**0.57 [0.43–0.76]**	1.04 [0.81–1.32]	0.94 [0.72–1.23]
AST [U/L]	1.36 [0.79–2.29]	1.00 [0.63–1.55]	1.16 [0.81–1.64]	1.30 [0.91–1.85]	0.89 [0.66–1.19]	0.91 [0.66–1.25]
ALP [U/L]	1.11 [0.71–1.74]	1.26 [0.91–1.75]	1.15 [0.87–1.51]	1.22 [0.93–1.59]	1.21 [0.97–1.50]	1.21 [0.95–1.53]
γGT [U/L]	**1.51 [1.21–1.86]**	**1.22 [1.03–1.44]**	**1.34 [1.17–1.53]**	**1.24 [1.08–1.42]**	**1.12 [1.00–1.26]**	1.00[0.88–1.13]
Bilirubin [µM]	1.18 [0.88–1.60]	0.95 [0.75–1.20]	1.04 [0.86–1.26]	0.87 [0.72–1.06]	1.06 [0.91–1.25]	1.01 [0.87–1.19]

Table entries are hazard ratios (HR) adjusted for sex, age, diabetes duration, body mass index, HbA_1C_, high-density lipoprotein cholesterol, low density lipoprotein cholesterol, estimated glomerular filtration rate, albumin/creatinine ratio, systolic blood pressure, serum albumin, cigarette smoking, prior CV disease, history of HF, and use of loop and/or non-loop diuretics, renin–angiotensin–aldosterone system inhibitors, statins, antithrombotics or beta-blockers. Statistically significant estimates are in bold

*ALT* alanine aminotransferase; *AST* aspartic aminotransferase; *γGT* gamma-glutamyl transferase; *ALP* alkaline phosphatase; *CV* cardiovascular; *HF* hospitalized heart failure; *MACE* major adverse cardiovascular events

^a^Renal endp. = composite of a > 40% decline in eGFR, renal replacement, renal death, and progression to macroalbuminuria

### Treatment effect

Over two years, canagliflozin treatment resulted in statistically significant decrements in serum ALT, ALP, and γGT (Additional file 1: Fig. S1) and, for γGT only, a significant decrease in the percentage of values ≥ 3*x*ULN. This decrement in ALT, ALP, and γGT was mainly apparent in the first year, subsequently reaching a plateau. AST was almost stable over time and bilirubin rose only slightly.

**Fig. 2:**
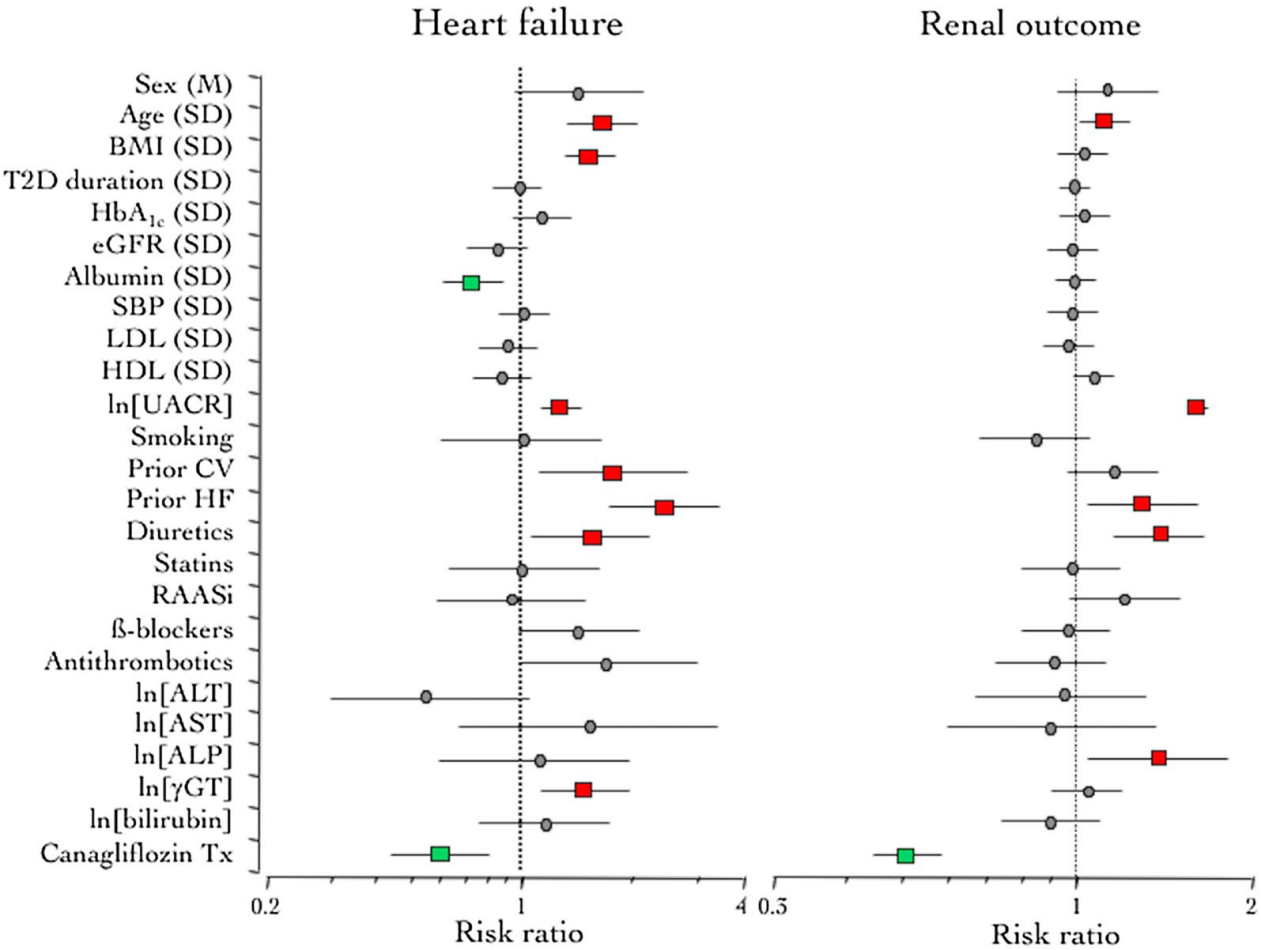
Multivariate Cox proportional hazards model of the association of updated LFTs, risk markers and canagliflozin treatment with incident hospitalized HF (left) and renal outcome (right). *M* male sex; *SD* standard deviation; *BMI* body mass index; *T2D* type 2 diabetes; *eGFR* estimated glomerular filtration rate; *SBP* systolic blood pressure; *HDL* high-density lipoprotein cholesterol; *LDL* low density lipoprotein cholesterol; *CV* cardiovascular; *HF* heart failure; *RAASi* renin angiotensin aldosterone system inhibitors; *ALT* alanine aminotransferase; *AST* aspartic aminotransferase; *ALP* alkaline phosphatase; *gGT* gamma-glutamyl transferase

Canagliflozin treatment alone was associated with marked relative risk reduction of CV outcomes—especially HF—and the renal endpoint (Additional file 2: Fig. S2), CV death falling short of statistical significance. By controlling for the 19 potential confounders listed in Additional file 4: Table S2, the effect of treatment on all-cause mortality was somewhat weakened (Additional file 4: Table S3). Therefore, when adjusting the effect of treatment by the set of 19 covariates used for the baseline LFTs and using updated LFTs values, i.e., the average LFTs level at weeks 52, 78 and 104, treatment was no longer associated with death, whether CV, CV combined with HF, or all-cause. Updated γGT remained a positive risk predictor of each CV outcome and all-cause death. Notably, in addition to updated γGT, age, BMI, UACR, prior CV and HF history, and use of diuretics were independent risk factors, while serum albumin was associated with a lower risk of HF hospitalization (Fig. 2). For the renal outcome, most risk predictors were the same as those for HF (age, UACR, prior HF, and use of diuretics), but updated ALP instead of updated γGT was one additional independent risk predictor (Fig. 2).

To test the performance of the AST/ALT ratio, we ran a separate set of Cox models with baseline or updated LFTs in which AST and ALT were replaced by their ratio. In both data sets, AST/ALT was an additional risk factor paralleling γGT in the prediction of HF hospitalization—alone or in combination with CV death—and all-cause death (Additional file 4: Table S4).

## Discussion

Our first general finding is the widespread association of the five primary LFTs with anthropometric and biochemical parameters as well as common drugs (Additional file 4: Table S2). These associations closely reproduce those from a previous large study including both patients with and without type 2 diabetes [10]. The underpinning of these relationships is heterogeneous, as transaminases are released by many different tissues in amounts that depend on physiological circumstances [13]. ALT, for example, declines with moderate weight loss (comparable to that achieved by SGLT2i therapy) [22], whereas AST is stable; therefore, ALT changes may equally reflect reduction of liver fat or decrease in lean body mass [10].

Because of the potential for strong confounding, the associations between baseline LFTs and individual outcomes were adjusted for a set of 19 covariates as well as all five LFTs. This analysis strategy caused the results to collapse to just ALT and γGT, the former carrying a reduced risk, and the latter an enhanced risk, for both HF hospitalization and death (CV and all-cause), neither being predictive of functional renal decline (Table 2). Of note, one or the other LFT has been repeatedly reported to be associated with prevalent or incident CVD [13, 15, 16, 18–21], but no previous study has carried out a systematic test of the relative predictive power of baseline LFTs for multiple adjudicated outcomes. In a comprehensive epidemiological meta-analysis involving over 9 million individuals, baseline γGT was unequivocally associated with excess all-cause mortality [24]. In the British Women’s Heart and Health study, γGT was associated with incident coronary artery disease and stroke independently of alcohol intake [25]. Potential mechanisms linking γGT with CV risk hinge upon oxidative stress, with glutathione consumption, direct reductant activity of the cysteinyl glycine residue of glutathione and, possibly, γGT–mediated redox reactions within the atherosclerotic plaque [13, 26]. In studies in patients undergoing bariatric surgery, the four γGT fractions resolved by fast protein-liquid chromatography have been shown to bear differential relation to obesity and insulin resistance [27].

In contrast to γGT, the interpretation of the inverse association of baseline ALT with CV outcomes is more challenging. High ALT levels are often a marker of non-alcoholic fatty liver disease in patients with type 2 diabetes, and therefore their reduction by SGLT2i therapy [28] would be expected to be associated with lower CV risk [29]. However, caution is required when interpreting mild ALT elevation, especially because it might derive from extra-hepatic sources [10, 12]. We may therefore postulate that, at least in type 2 diabetes subjects at high CV risk such as the CANVAS Program population, baseline ALT largely reflect lean body mass (*cfr*., the positive correlation with BMI, Additional file 4: Table S1), the relative scarcity of which (sarcopenia) is an obesity-independent risk factor for CVD [30].

In univariate analysis, randomized treatment with canagliflozin was associated with relative risk reductions of first MACE, HF hospitalization—with or without CV death—all-cause mortality, and renal dysfunction, as reported previously [22]. Adjusting the univariate HRs for the set of 19 confounders—which included background therapies—weakened the impact of canagliflozin treatment on CV outcomes only marginally, thus attesting to a good performance of randomization in the CANVAS Program. Treatment also led to consistent, if small, decrements in baseline serum ALT, γGT, and ALP concentrations, especially in the first year since randomization (Additional file 1: Fig. S1). This might be attributable to the overall improvement of the metabolic profile with canagliflozin treatment. In CANVAS, several other intermediate markers of cardiovascular risk, including HbA1c, body weight and blood pressure, decreased during the first 52 weeks of treatment (Fig. 1 ref. [22]). Consistently, in a pooled analysis of six phase III trials, the significant decrease in serum LFT in the canagliflozin treatment arms was statistically fully explained by the combined effects on body weight and HbA1c [31]. The positive risk association of γGT with all CV events and the inverse risk association of ALT with HF hospitalization/CV death and total mortality were maintained when post-randomization LFT values were included in the prediction model; updated ALP emerged as an independent risk for progression of renal dysfunction (Fig. 2). Treatment per se, however, was no longer associated with a reduction in the relative risk of outcomes that include death (i.e., MACE, CV death, HF hospitalization/CV death, and all-cause death). Thus, the incorporation of LFTs into the prediction model led to a partitioning of risk between canagliflozin and the LFTs as mediators of the impact of treatment on each outcome. Conceptually, this data analysis is akin to mediation analysis, which has been done with canagliflozin, separately for HF [32] and the renal outcome [33], and with empagliflozin for CV mortality [34]. However, we chose not to perform formal mediation analyses of individual outcomes because we were rather interested in examining the potential predictive role of LFTs as ubiquitous biomarkers across all adjudicated outcomes and to maximize information about them as intermediate factors in the risk reduction observed with canagliflozin (i.e., indirect perspectiveness) [35]. Also, formal mediation analysis is known to be fraught with interpretative difficulties, particularly when testing multiple mediators—which may be collinear or have opposite sign—without a specific hypothesis [35].

Eventually, our main finding is that the risk of death was partitioned to raised γGT levels, while canagliflozin protected against HF independently of a broad set of potential confounders. That γGT in the accepted safety range would stand out as such a powerful risk factor was partly unexpected. Also, γGT is unlikely to be a bona fide mediator as treatment reduced its levels by only 5 (35%) (Fig. 2), and the positive association of γGT with deaths was also evident in the baseline, pre-treatment dataset. Our results support the proposed underlying pathophysiological mechanism that γGT might be a proatherogenic marker through LDL oxidation [17], as detailed in Additional file 3: Fig. S3; indeed, enzyme activity has been found in coronary atheroma from surgical specimens [36].

The clean segregation of the risk of death within LFTs supports the contention that the ‘pure’ cardiac benefit of SGLT2i is on HF-related events, the effect on mortality being quite variable across trials [8].

The pattern of risk predictors for the renal endpoint mirrored the one for HF, except that ALP replaced γGT in the multi-adjusted post-randomization model. ALP is a ubiquitous enzyme that has been associated with CVD and CKD via multiple mechanisms, including vascular calcification, oxidative stress, and inflammation [37, 38]. Particularly, ALP is involved in tissue mineralization, which is dysregulated in CKD and might ultimately lead to CKD progression and mortality, as thoroughly reviewed by Haarhaus and colleagues [37], who proposed ALP as a potential treatment target. On the other hand, it has been suggested that SGLT2i might have detrimental effects on bone metabolism: the inhibition of sodium reabsorption leads to increased parathyroid hormone levels and reduced 1,25-dihydroxy vitamin D levels, impairing skeletal mineralization, and the induced weight loss might indirectly increase bone turnover [39]. The clinical impact of such alterations is debated: an increased risk of fractures was reported in CANVAS [40] but it was not confirmed in other studies investigating the effect of canagliflozin nor was it observed in large meta-analyses on SGLT2i [39], thus suggesting that it is possibly related to extrinsic factors, such as higher occurrence of falls and overall frailty of the CANVAS population. Even though we did not investigate fractures in the present analysis, our findings seem to confirm that there is a significant impact of SGLT2 inhibition on ALP levels, possibly reflecting the interplay with bone metabolism, thus at least partly explaining the specific association with renal endpoints.

A final consideration concerns the AST/ALT ratio, the precise clinical significance of which remains vague when values are within the normal range, despite its widespread use as a marker of acute and chronic liver dysfunction. The fact that in the multi-adjusted post-randomization model it tracked with γGT as an indicator of CV risk (Additional file 4: Table S4) likely stands for a degree of liver dysfunction participating in the overall CV risk of the CANVAS population. Consistently, an association between AST/ALT ratio within the normal range and increased risk of all-cause and CV mortality has been previously reported in unselected populations with diabetes [41] and hypertension [42].

This analysis has several strengths, including the high quality and internal validity of the data set, deriving from a large CVOT, the number of biomarkers available for analysis, and the repeated measures that allowed to explore associations prospectively.

Some limitations must be acknowledged. First, this is a post hoc investigation and as such its robustness is limited, given multiple testing. Second, all trial participants had been selected at inclusion as having LFTs within the normal reference values, therefore limiting our findings to this baseline range. Another limitation of our analysis is that the exact distribution of weights among LFTs may be unstable as it depends on the precision of the respective assays or other experimental circumstances. Moreover, despite adjusting for a high number of confounders, it is possible that additional significant confounders were not captured. Finally, biomarker domains identified in previous post hoc analyses of CANVAS (e.g., hematocrit, urate, hemoglobin, NTproBNP [33, 34, 43]) were not included in the current analysis, which circumscribed the working hypothesis to the role of LFTs.

## Conclusions

In conclusion, in the CANVAS trial higher baseline and post-randomization γGT levels were independently predictive of the risk of death, while canagliflozin protected against HF hospitalization and renal damage independently of LFTs and a broad set of potential confounders. Future exploration of other CVOTs may help complete the picture of CV and renal risk in individuals with or without diabetes and offer further pathogenetic and therapeutic clues.

## Supplementary Information

**Additional file 1: Figure S1.** Time-course of ALT, γGT, and ALP concentrations in the treatment (canagliflozin) and placebo arm. The *p* values are for the difference between the two arms by repeated-measures ANOVA. Plots are mean ± SD. *ALT* alanine aminotransferase; *AST* aspartic aminotransferase; *ALP* alkaline phosphatase; *gGT* gamma-glutamyl transferase.

**Additional file 2: Figure S2. a** Kaplan–Meier plot of time to first hospitalized heart failure by treatment; **b** Kaplan–Meier plot of time to renal endpoint by treatment.

**Additional file 3: Figure S3.** Steps in the gamma glutamyl transferase enzyme reaction and its relationship to the oxidation of low-density lipoprotein cholesterol. Reproduced from Mason et al. ref. [17] with permission from Wiley´s permission department.

**Additional file 4: Table S1.** LFT levels at baseline and 2 years by treatment*. **Table S2.** Univariate association (Spearman’s *r* or Wilcoxon test) of baseline LFTs with clinical characteristics. **Table S3.** Association with CV and renal outcomes (HR [95% C.I.]) of canagliflozin treatment alone, treatment adjusted for the covariates,* and treatment further adjusted for 5 updated LFTs. **Table S4.** Multivariate association (HR [95% CI]) of AST/ALT + treatment with CV and renal outcomes*.

## Data Availability

The data that support the findings of this study are available from Janssen Research and Development, LLC but restrictions apply to the availability of these data, which were used under license for the current study, and so are not publicly available. Data are however available from the authors upon reasonable request and with permission of Janssen Research and Development, LLC.

## Abbreviations

ASCVD: Atherosclerotic cardiovascular disease
ALP: Alkaline phosphatase
ALT: Alanine aminotransferase
AST: Aspartic aminotransferase
BMI: Body mass index
BP: Blood pressure
CANVAS: Canagliflozin Cardiovascular Assessment Study
CKD: Chronic kidney disease
CV: Cardiovascular
CVD: Cardiovascular disease
CVOT: Cardiovascular outcome trials
eGFR: Estimated glomerular filtration rate
HbA_1c_: Glycated hemoglobin A_1C_
HF: Heart failure
HHF: Hospitalized heart failure
LFTs: Liver function tests
MACE: Major adverse cardiac events
RAAS: Renin–angiotensin–aldosterone-system
SGLT2i: Sodium-glucose cotransporter 2 inhibitors
UACR: Urinary albumin-to-creatinine ratio
ULN: Upper limit of normal
γGT: Gamma-glutamyl transferase
