# Supplementary figures and images for: Liver function markers predict cardiovascular and renal outcomes in the CANVAS Program
Source: Cardiovasc Diabetol. 2022 Jul 4;21:127. doi: 10.1186/s12933-022-01558-w (PMC9254689; doi:10.1186/s12933-022-01558-w)

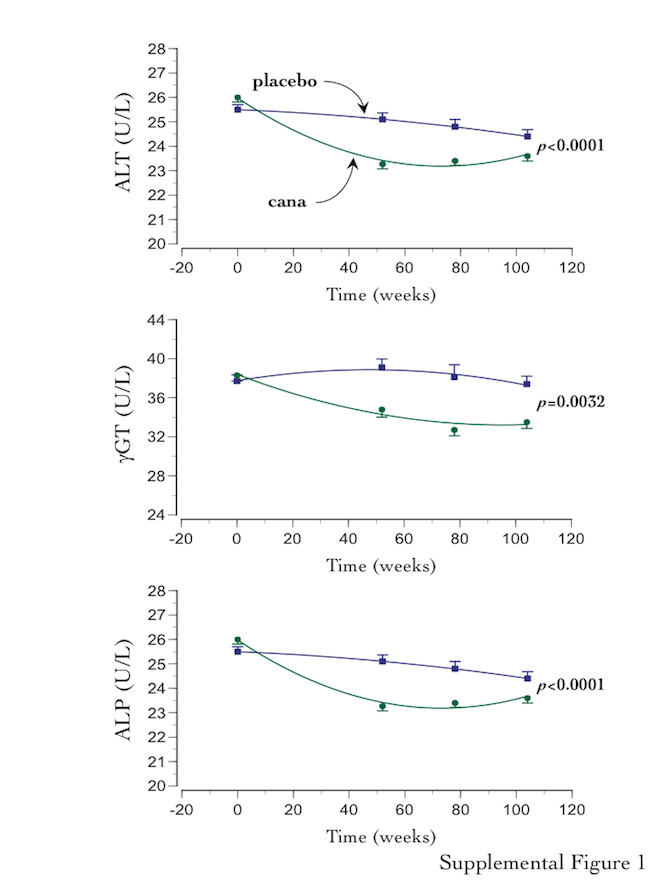

Supplement: Supplementary file 1 — Additional file 1: Figure S1. Time-course of ALT, γGT, and ALP concentrations in the treatment (canagliflozin) and placebo arm. The p values are for the difference between the two arms by repeated-measures ANOVA. Plots are mean ± SD. ALT alanine aminotransferase; AST aspartic aminotransferase; ALP alkaline phosphatase; gGT gamma-glutamyl transferase. [file 12933_2022_1558_MOESM1_ESM.tiff]

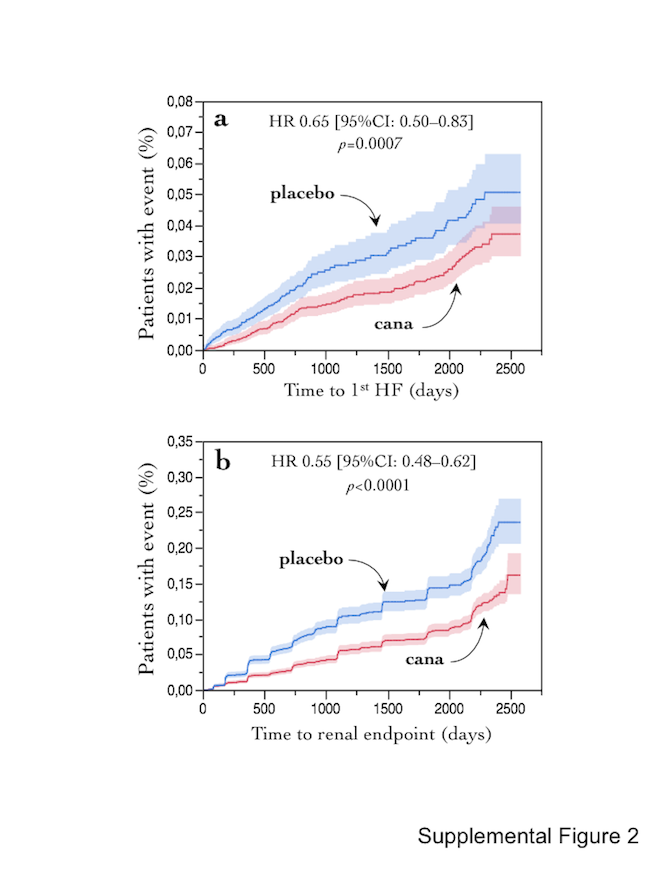

Supplement: Supplementary file 2 — Additional file 2: Figure S2. a Kaplan–Meier plot of time to first hospitalized heart failure by treatment; b Kaplan–Meier plot of time to renal endpoint by treatment. [file 12933_2022_1558_MOESM2_ESM.tiff]

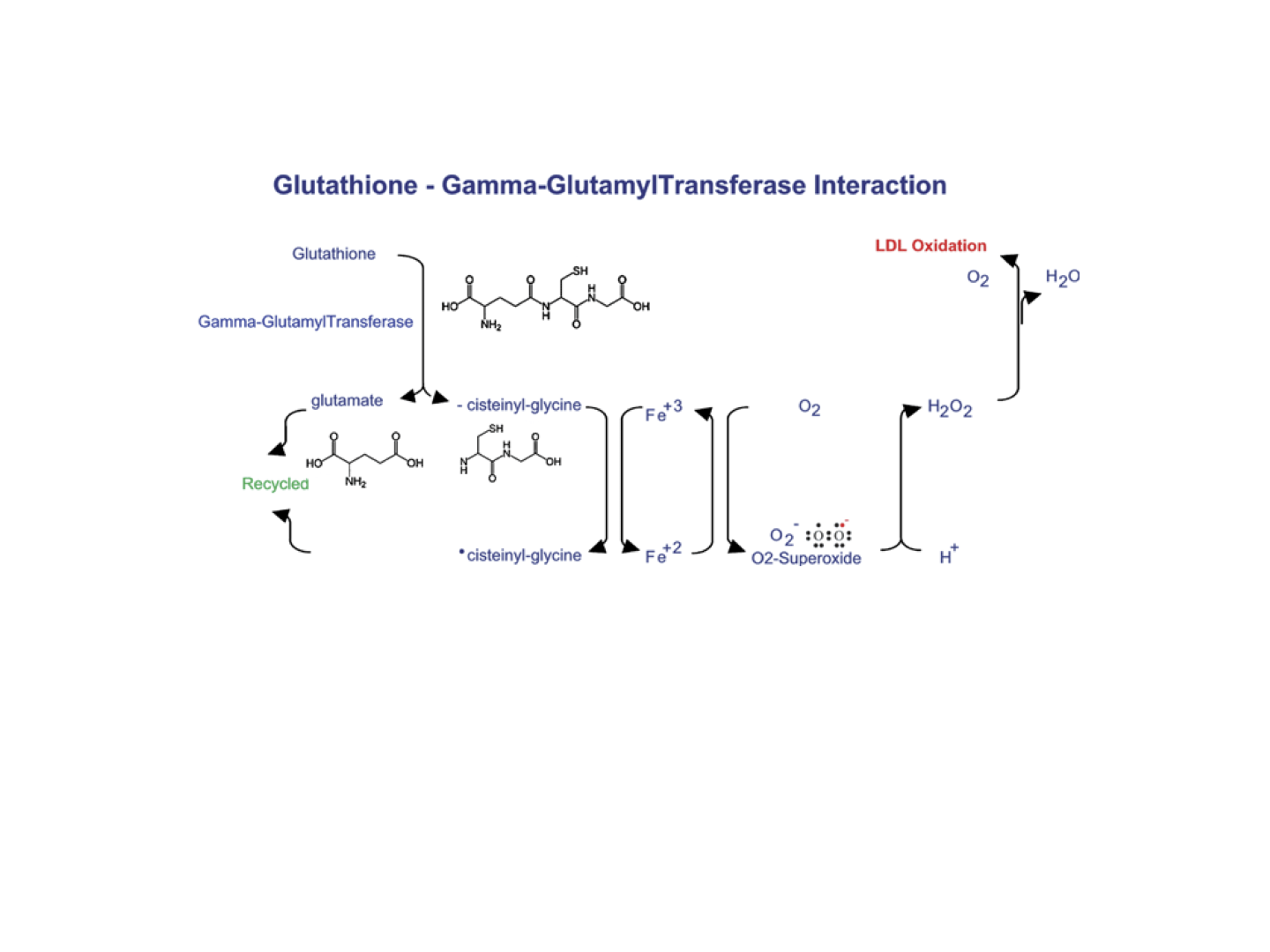

Supplement: Supplementary file 3 — Additional file 3: Figure S3. Steps in the gamma glutamyl transferase enzyme reaction and its relationship to the oxidation of low-density lipoprotein cholesterol. Reproduced from Mason et al. ref. [17] with permission from Wiley´s permission department. [file 12933_2022_1558_MOESM3_ESM.tiff]
