# Supplementary material for: Liver function markers predict cardiovascular and renal outcomes in the CANVAS Program
Source: Cardiovasc Diabetol. 2022 Jul 4;21:127. doi: 10.1186/s12933-022-01558-w (PMC9254689; doi:10.1186/s12933-022-01558-w)
Supplement: Supplementary file 4 — Additional file 4: Table S1. LFT levels at baseline and 2 years by treatment*. Table S2. Univariate association (Spearman’s r or Wilcoxon test) of baseline LFTs with clinical characteristics. Table S3. Association with CV and renal outcomes (HR [95% C.I.]) of canagliflozin treatment alone, treatment adjusted for the covariates,* and treatment further adjusted for 5 updated LFTs. Table S4. Multivariate association (HR [95% CI]) of AST/ALT + treatment with CV and renal outcomes*. [file 12933_2022_1558_MOESM4_ESM.docx]

**Table S1 – LFT levels at baseline and 2 years by treatment.***

|  | **Placebo (n=4,276)** | | | | **Treatment (n=5,710)** | | | |
| --- | --- | --- | --- | --- | --- | --- | --- | --- |
|  | **Baseline** | | **2 years** | | **Baseline** | | **2 years** | |
|  | **Mn [IQR]** | **% ≥3*x*ULN** | **Mn [IQR]** | **% ≥3*x*ULN** | **Mn [IQR]** | **% ≥3*x*ULN** | **Mn [IQR] [IQR][IQR][IQR][IQR]** | **% ≥3*x*ULN** |
| **ALT (U/L)** | 22 [15] | 0.14 | 21 [13] | 0.11 | 22 [14] | 0.12 | 21 [11] | 0.12 |
| **AST (U/L)** | 20 [10] | 0.07 | 19 [8] | 0.08 | 20 [9] | 0.12 | 19 [8] | 0.09 |
| **ALP (U/L)** | 73 [29] | 0 | 72 [29] | 0.03 | 73 [29] | 0.02 | 72 [29] | 0.04 |
| **γGT (U/L)** | 26 [22] | 1.99 | 25 [22] | 2.43^†^ | 27 [23] | 2.36 | 23 [18] | 1.75^†^ |
| **Bilirubin (µM)** | 8 [4] | 0 | 7 [5] | 0 | 8 [4] | 0 | 8 [4] | 0.03 |

*Mn [IQR] = median [interquartile range]; % ≥3*x*ULN = percentage of values exceeding 3 upper limits of normal. ALT = alanine aminotransferase; AST = aspartic aminotransferase; ALP = alkaline phosphatase; γGT = gamma-glutamyl transferase;

^†^ *p*<0.0001 *vs* baseline.

**Table S2 – Univariate association (Spearman’s *r* or Wilcoxon test] of baseline LFTs with clinical characteristics.**

|  | **ALT** | **AST** | **ALP** | **γGT** | **Bilirubin** | **Albumin** | **AST/ALT** |
| --- | --- | --- | --- | --- | --- | --- | --- |
| **ALT (U/L)** | . | . | . | . | . | . | . |
| **AST (U/L)** | 0.77 | . | . | . | . | . | . |
| **ALP (U/L)** | -0.03 | -0.03 | . | . | . | . | . |
| **γGT (U/L)** | 0.49 | 0.41 | 0.14 | . | . | . | . |
| **Bilirubin (µmol/L)** | 0.14 | 0.16 | -0.03 | 0.07 | . | . | . |
| **Serum albumin (g/L)** | 0.19 | 0.15 | -0.15 | 0.09 | 0.18 | . | . |
| **AST/ALT** | -0.64 | -0.06 | ns | -0.26 | -0.03 | -0.14 | . |
| **Sex (M)** | + | + | – | + | + | + | – |
| **Age (years)** | -0.21 | -0.06 | -0.04 | -0.15 | 0.06 | -0.11 | 0.26 |
| **BMI (kg/m^2^)** | 0.14 | 0.08 | ns | 0.19 | -0.07 | -0.19 | -0.12 |
| **T2D duration (years)** | -0.13 | -0.05 | -0.04 | -0.17 | -0.06 | -0.13 | 0.15 |
| **HbA_1c_ (%)** | 0.05 | ns | 0.14 | 0.11 | -0.04 | -0.07 | -0.09 |
| **HDL (mg/dL)** | -0.12 | -0.04 | 0.03 | -0.11 | ns | ns | 0.14 |
| **LDL (mg/dL)** | -0.05 | -0.06 | 0.10 | 0.04 | -0.06 | ns | ns |
| **Systolic BP (mmHg)** | 0.03 | ns | 0.02 | 0.04 | -0.02 | ns | ns |
| **eGFR (mL^.^min^-1.^1.73m^-2^)** | 0.11 | ns | ns | 0.07 | 0.04 | 0.07 | -0.19 |
| **UACR (mg/g)** | -0.06 | ns | 0.07 | 0.06 | -0.08 | -0.12 | 0.08 |
| **Smoking (yes *vs* no)** | – | – | + | + | – | ns | – |
| **Prior CVD (yes *vs* no)** | – | ns | ns | + | + | + | + |
| **History of HF (yes *vs* no)** | – | – | + | + | + | ns | + |
| **Diuretics (yes *vs* no)** | ns | + | – | + | ns | - | + |
| **RAAS inhibitors (yes *vs* no)** | + | ns | – | + | – | ns | – |
| **Statins (yes *vs* no)** | + | + | – | ns | ns | + | – |
| **Antithrombotics (yes *vs* no)** | ns | + | – | ns | + | + | + |
| **ß-blockers (yes *vs* no)** | + | + | – | + | + | ns | + |

M = male sex; BMI = body mass index; T2D = type 2 diabetes; HDL = high-density lipoprotein cholesterol; LDL = low density lipoprotein cholesterol; BP = blood pressure; eGFR = estimated glomerular filtration rate; UACR = urinary albumin-to-creatinine ratio; CVD = cardiovascular disease; HF = heart failure; RAAS = renin angiotensin aldosterone system.

**Table S3 – Association with CV and renal outcomes (HR [95% C.I.]] of canagliflozin treatment alone, treatment adjusted for the covariates,* and treatment further adjusted for 5 updated LFTs.**

|  | **HF** | **CV Death** | **HF or CV Death** | **All Deaths** | **MACE** | **Renal endp.**^†^ |
| --- | --- | --- | --- | --- | --- | --- |
| **Treatment alone** | **0.65 [0.50–0.83]** | 0.87 [0.72–1.06] | **0.77 [0.66–0.90]** | **0.86 [0.73-1.00]** | **0.85 [0.75–0.97]** | **0.55 [0.48–0.62]** |
| **Adj. Treatment** | **0.65 [0.50–0.84]** | 0.89 [0.74–1.08] | **0.78 [0.67–0.91]** | 0.87 [0.74–1.02] | **0.87 [0.75–0.97]** | **0.55 [0.48–0.63]** |
| ***Log [updated]*** |  |  |  |  |  |  |
|  |  |  |  |  |  |  |
| **ALT (U/L]** | 0.56 [0.30–1.07] | 0.59 [0.31–1.13] | **0.52 [0.32–0.82]** | **0.49 [0.30–0.82]** | 1.19 [0.82–1.73] | 0.95[0.67–1.34] |
| **AST (U/L]** | 1.54 [0.68–3.39] | 0.57 [0.23–1.34] | 1.01 [0.55–1.84] | 0.78 [0.39–1.54] | **0.59 [0.36–0.94]** | 0.90 [0.59–1.38] |
| **ALP (U/L]** | 1.12 [0.61–2.04] | 0.97 [0.53–1.77] | 1.11 [0.72–1.72] | 1.16 [0.72–1.87] | 1.22 [0.89–1.66] | **1.38 [1.04–1.84]** |
| **γGT (U/L]** | **1.51 [1.14–2.00]** | **1.49 [1.09–2.00]** | **1.49 [1.20–1.84]** | **1.55 [1.21–1.96]** | **1.19 [1.01–1.39]** | 1.04 [0.90–1.20] |
| **Bilirubin (µM]** | 1.16 [0.77–1.76] | 0.93 [0.60–1.42] | 1.09 [0.80–1.48] | 0.87 [0.62–1.23] | 1.05 [0.84–1.31] | 0.90 [0.73–1.11] |
| **Adj. Treatment+LFTs** | **0.60 [0.43­–0.84]** | 1.14 [0.81–1.64] | 0.79 [0.62-1.01] | 0.99 [0.75–1.30] | 0.90 [0.76–1.08] | **0.51 [0.43–0.59]** |

*Adj. = hazard ratio adjusted for: sex, age, diabetes duration, body mass index, HbA_1C_, high-density lipoprotein cholesterol, low density lipoprotein cholesterol, estimated glomerular filtration rate, albumin/creatinine ratio, systolic blood pressure, serum albumin, daily cigarette smoker, prior CV disease, history of HF, use of loop and/or non-loop diuretics, RAAS inhibitors, statins, antithrombotics or beta-blockers.

ALT = alanine aminotransferase; AST = aspartic aminotransferase; γGT = gamma-glutamyl transferase; ALP = alkaline phosphatase; CV = cardiovascular; HF = hospitalized heart failure; MACE = major adverse cardiovascular events.

^†^Renal endp. = composite of a >40% decline in eGFR, renal replacement, renal death, and progression to macroalbuminuria.

**Table S4 – Multivariate association (HR [95% CI]] of AST/ALT + treatment with CV and renal outcomes.***

|  | **HF** | **CV Death** | **HF or CV Death** | **All Deaths** | **MACE** | **Renal endp.**^†^ |
| --- | --- | --- | --- | --- | --- | --- |
| ***Log [baseline]*** |  |  |  |  |  |  |
| **ALP (U/L]** | 1.16 [0.74–1.80] | 1.30 [0.93–1.80] | 1.19 [0.90–1.56] | 1.25 [0.96–1.63] | 1.22 [0.98–1.51] | 1.22 [0.97–1.55] |
| **γGT (U/L]** | **1.38 [1.14–1.66]** | 1.12 [0.96–1.30] | **1.22 [1.08–1.37]** | **1.15 [1.02–1.30]** | 1.10 [1.00–1.22] | 0.96 [0.86–1.07] |
| **Bilirubin (µM]** | 1.16 [0.86–1.57] | 0.93 [0.74–1.17] | 1.01 [0.84–1.23] | 0.86 [0.71–1.03] | 1.06 [0.91–1.23] | 1.01 [0.86–1.18] |
| **AST/ALT** | **1.73 [1.11–2.66]** | 1.39 [0.97–1.97] | **1.57 [1.18–2.08]** | **1.67 [1.26–2.21]** | 0.95 [0.75–1.21] | 1.04 [0.80–1.34] |
| ***Log [updated]*** |  |  |  |  |  |  |
| **ALP (U/L]** | 1.12 [0.61–2.04] | 0.97 [0.53–1.77] | 1.14 [0.74–1.76] | 1.23 [0.77–1.98] | 1.25 [0.92–1.71] | **1.39 [1.05–1.85]** |
| **γGT (U/L]** | **1.46 [1.14–1.86]** | 1.18 [0.90–1.54] | **1.29 [1.07–1.55]** | **1.25 [1.01–1.54]** | 1.09 [0.95–1.26] | 1.01 [0.89–1.16] |
| **Bilirubin (µM]** | 1.15 [0.76–1.74] | 0.88 [0.57–1.36] | 1.06 [0.77–1.44] | 0.83 [0.59–1.17] | 1.02 [0.82–1.27] | 0.90 [0.73–1.10] |
| **AST/ALT** | **2.01 [1.06–3.68]** | 1.61 [0.84–2.99] | **1.99 [1.26–3.10]** | **1.87 [1.11–3.07]** | 0.84 [0.58–1.21] | 1.13 [0.80–1.60] |
| **Adj. Treatment** | **0.60 [0.43­–0.84]** | 1.14 [0.81–1.63] | 0.78 [0.62-1.00] | 0.99 [0.75–1.30] | 0.90 [0.76–1.08] | **0.51 [0.43–0.59]** |

*Hazard ratios adjusted for sex, age, diabetes duration, BMI, HbA_1C_, HDL, LDL, eGFR, albumin/creatinine ratio, systolic blood pressure, serum albumin, daily cigarette smoker, prior CV disease, history of HF, use of loop and/or non-loop diuretics, RAAS inhibitors, statins, antithrombotics or beta-blockers; ALP = alkaline phosphatase; γGT = gamma-glutamyl transferase; AST = aspartic aminotransferase; ALT = alanine aminotransferase; HF = hospitalized heart failure; CV = cardiovascular; MACE = major adverse cardiovascular events.

^†^Renal endp. = composite of a >40% decline in eGFR, renal replacement, renal death, and progression to macroalbuminuria.
